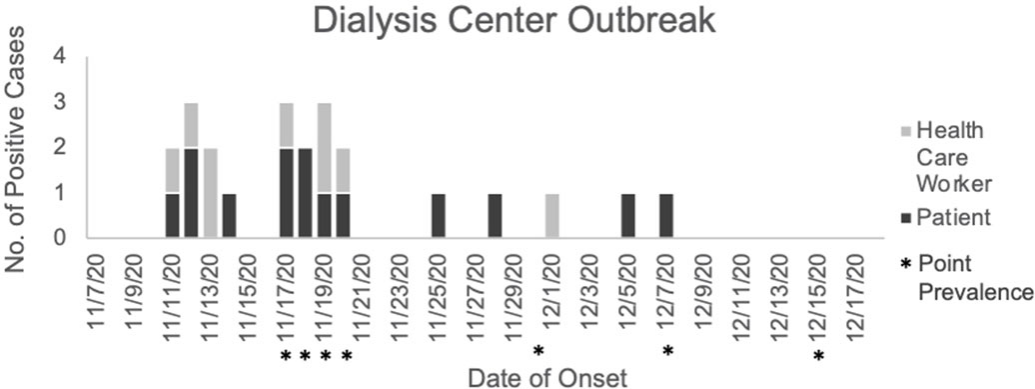# Characteristics of On-Site Infection Prevention and Control Visits for COVID-19—California, February 2020–December 2020

**DOI:** 10.1017/ash.2021.95

**Published:** 2021-07-29

**Authors:** Patrick Stendel, Ellora Karmarkar, Idamae Kennedy, Hosniyeh Bagheri, Teresa Nelson, Terry Nelson, Erin Epson

## Abstract

**Background:** The novel coronavirus (COVID-19) pandemic has caused significant morbidity and mortality in California: 2,218,000 cases and 24,598 deaths had occurred by December 31, 2020. Deaths at skilled nursing facilities (SNFs) and assisted living facilities (ALFs) comprise 26.2% of deaths in California; the fatality rate (299 per 10,000 SNF and ALF residents) in such facilities is nearly 50 times the statewide COVID-19 mortality rate (6.4 per 10,000 California residents). For healthcare facility (SNF, ALF, acute-care hospitals) and correctional facility outbreak management, the California Department of Public Health (CDPH) Healthcare-Associated Infections (HAI) Program deployed trained infection preventionists (IPs) to perform on-site infection prevention and control (IPC) assessments and to provide recommendations to staff and local health departments (LHDs). We describe the number and distribution of visits across the state and common IPC challenges identified. **Methods:** From February 1, 2020, to December 31, 2020, CDPH IP visits were requested directly by facilities, coordinated through LHDs and other state agencies, or prompted by a facility’s increasing case count on twice weekly review of the daily California healthcare facility data survey (Survey 123). Deployed IPs evaluated facility COVID-19 IPC protocols, assessed facility staff adherence using a standardized assessment tool, and provided verbal feedback followed by written summary reports and recommendations. We categorized visits geographically into 5 California Health Officer Association regions and by month, and we reviewed visit reports for common findings. **Results:** In total, 623 visits were performed for 489 outbreaks at 465 distinct facilities across 46 LHDs; 71 facilities received ≥2 visits. Southern California facilities received 292 visits (46.9%), San Joaquin region facilities received 138 visits (22.2%), Bay Area facilities received 131 visits (21%), Greater Sacramento facilities received 54 visits (8.7%), and Rural North facilities received 8 visits (1.3%) (Figure 1). The highest number of visits per month occurred in December (n = 143, 22.9%), followed by July (n = 87, 13.9%), and April (n = 83, 13.3%). Common IPC challenges included inappropriate resident cohorting practices, improper use of personal protective equipment, and lapses in physical distancing, and source control in breakrooms. **Conclusions:** On-site visits by CDPH IPs during the COVID-19 pandemic in California, though resource-intensive, provided substantial technical support for healthcare facilities during outbreaks and identified key areas for IPC improvement. Ongoing CDPH HAI guidance and training materials for facility-based IP staff are now being informed by these IPC challenges.

**Funding:** No

**Disclosures:** None

**Figure 1.**
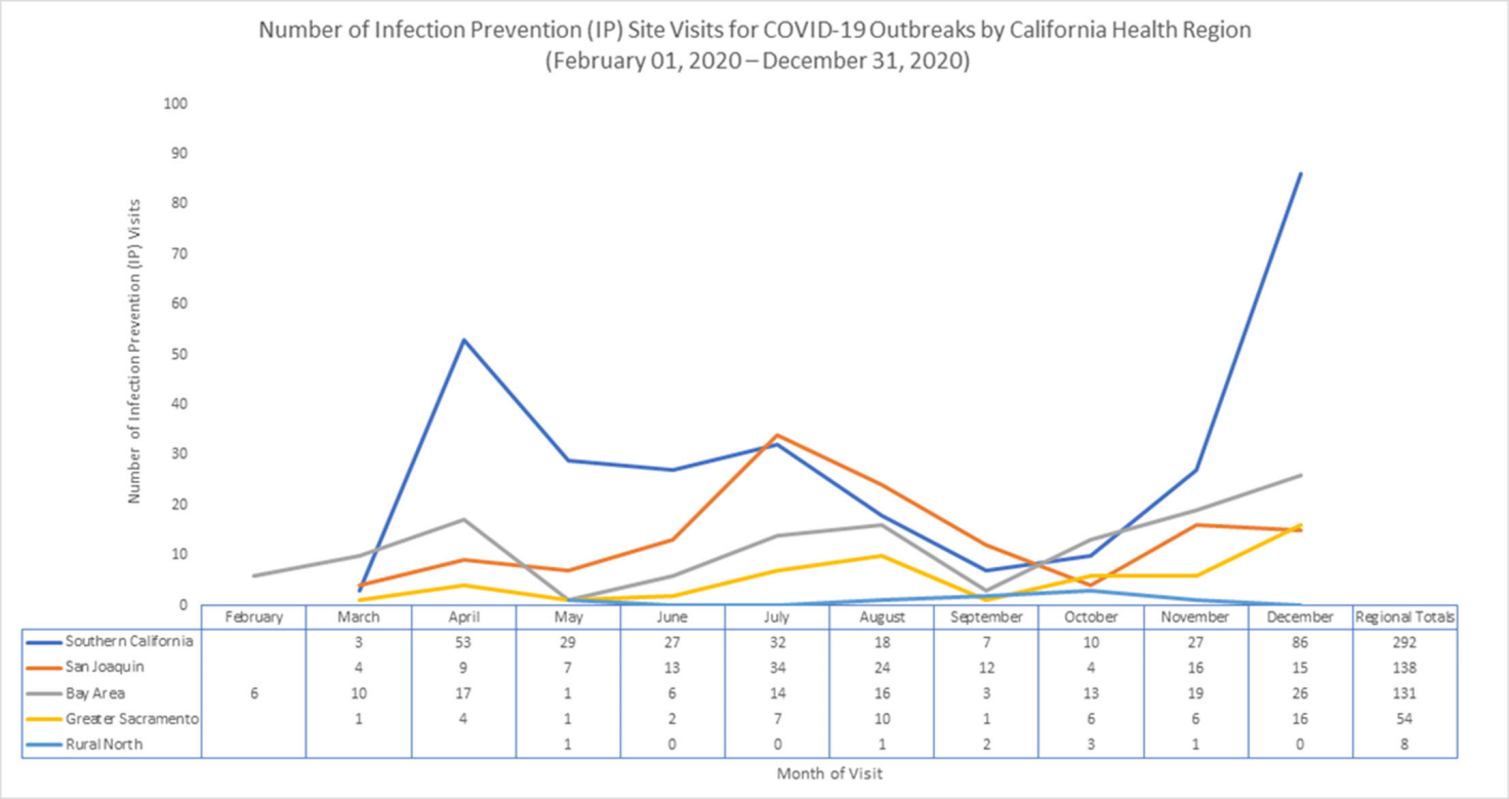

**Figure 2.**